# Crystal structure of the 1:1 adduct of (*E*)-5-(2,3-di­hydro­benzo[*d*]thia­zol-2-yl­idene)-2,6-dioxo-4-phenyl-1,2,5,6-tetra­hydro­pyridine-3-carbo­nitrile and its piperidinium salt, piperidinium (*Z*)-5-(benzo[*d*]thia­zol-2-yl)-3-cyano-6-oxo-4-phenyl-1,6-di­hydro­pyridin-2-olate

**DOI:** 10.1107/S2056989025006991

**Published:** 2025-08-12

**Authors:** Galal H. Elgemeie, Nadia H. Metwally, El-shimaa S. M. Abd Al-latif, Peter G. Jones

**Affiliations:** a Chemistry Department, Faculty of Science, Helwan University, Cairo, Egypt; b Chemistry Department, Faculty of Science, Cairo University, Giza, Egypt; c Institut für Anorganische und Analytische Chemie, Technische Universität Braunschweig, Hagenring 30, D-38106 Braunschweig, Germany; Universität Greifswald, Germany

**Keywords:** benzo­thia­zole, hydrogen bonds, crystal structure

## Abstract

In the structure of the title compound, C_5_H_12_N^+^·C_19_H_10_N_3_O_2_S^−^·C_19_H_11_N_3_O_2_S, the central pyridinic rings are approximately coplanar to the benzo­thia­zole moieties. The phenyl groups are appreciably angled to the central rings [inter­planar angles of 57.30 (3)° for the anion and 79.01 (4)° for the neutral mol­ecule]. Bond lengths and angles correspond to considerable delocalization of the π bonding, especially for the anion; all four C=O bond lengths are similar [1.2365 (13)–1.2591 (13) Å]. The two main residues display different configurations about the formally double C—C bonds between the benzo­thia­zole and pyridinic ring systems; the neutral mol­ecule is *E*, facilitating an intra­molecular N—H⋯O hydrogen bond, but the anion is *Z*, allowing a short intra­molecular S⋯O contact of 2.5794 (10) Å. Within the asymmetric unit, the piperidinium cation is hydrogen bonded to an oxygen atom of the anion; the anion and the neutral mol­ecule are connected by two N—H⋯O hydrogen bonds, forming a ring of graph-set *R*^2^_2_(8). Asymmetric units are linked to form inversion-symmetric dimers by an H_cation_⋯O_anion_ hydrogen bond. These are further linked by a C—H⋯O hydrogen bond to form a broad ribbon of residues parallel to the *a* axis.

## Abstract 2

The central pyridinic rings are approximately coplanar to the benzo­thia­zole moieties in both the neutral mol­ecule and the anion. Bond lengths and angles indicate considerable delocalization of the multiple bonding. The neutral mol­ecule is *E*-configured about the central C=C bond, but the anion is *Z*. Classical and ‘weak’ hydrogen bonds lead to a broad ribbon of residues.

## 1. Chemical context

**Figure 1:**
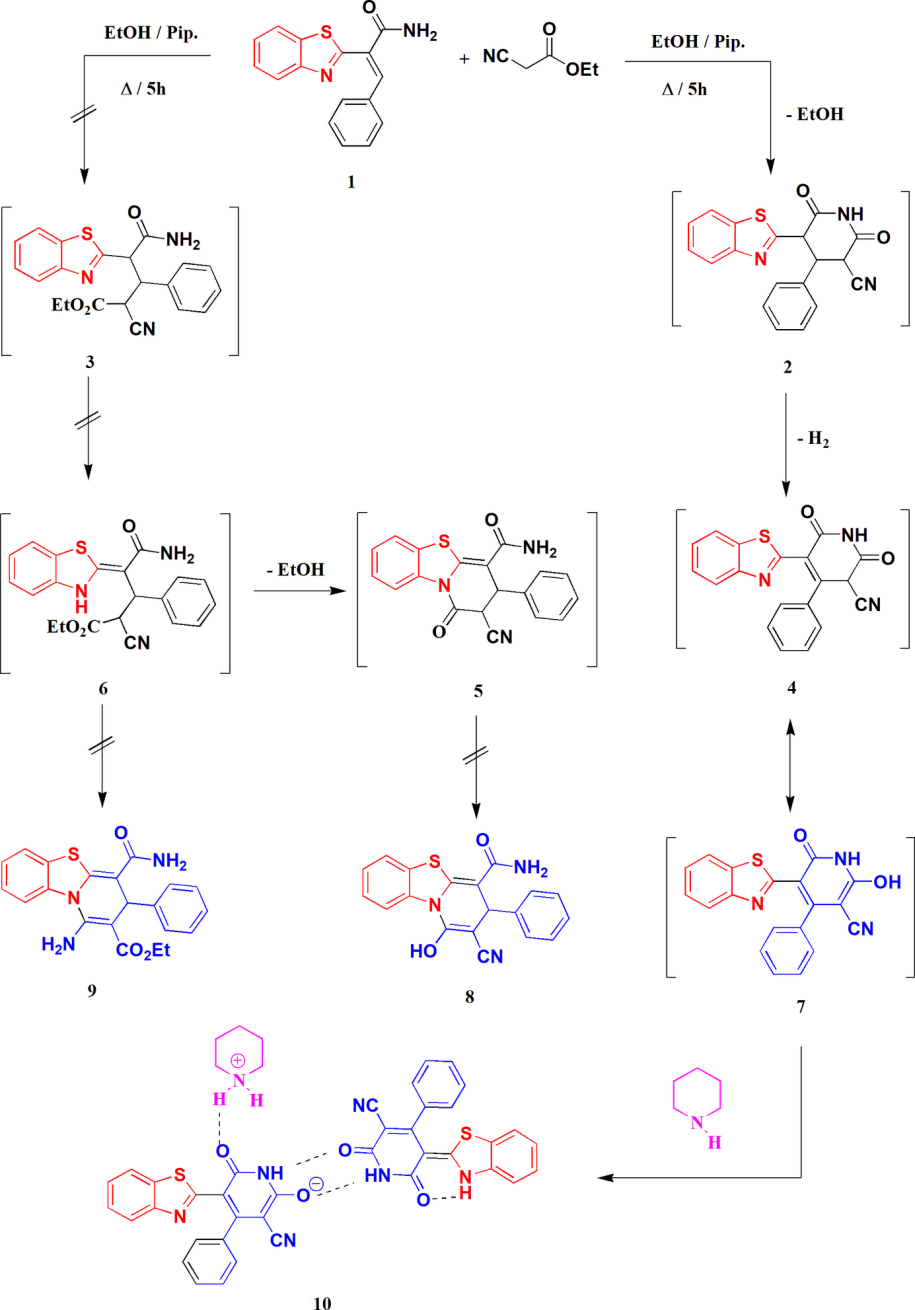
The reaction scheme for the synthesis of adduct **10**.

A wide range of pharmacological preparations contain benzo­thia­zoles, which are adaptable heterocyclic biologically active compounds (Azzam *et al.*, 2017 ▸; Elboshi *et al.*, 2024 ▸). Because of their exceptional pharmacological potential, these mol­ecules are very significant in the field of medicinal chemistry (Keri *et al.*, 2015 ▸). The hunt for novel therapeutic agents has benefited from the great degree of chemical variety displayed by benzo­thia­zole derivatives (Gill *et al.*, 2015 ▸). Since several benzo­thia­zole-based compounds have been utilized extensively as clinical medications to treat a variety of disorders with great therapeutic benefit, research in benzo­thia­zole-based medicinal chemistry has quickly become an important area (Sharma *et al.*, 2013 ▸). Medicinal chemists have invented numerous new synthetic methods targeting benzo­thia­zole-related derivatives (Azzam *et al.*, 2022 ▸; Elgemeie *et al.*, 2000 ▸). 2-Pyridyl­benzo­thia­zoles and 2-pyrim­id­in­yl­benzo­thia­zoles have emerged as a significant class of pharmacological agents in the creation of anti-tumour treatments in recent years (Azzam *et al.*, 2020 ▸; Das *et al.*, 2003 ▸); their synthetic accessibility and promising biological profile have aided in their development as possible chemotherapeutics. Many new synthetic techniques have been developed to introduce diversity and obtain this class of compounds in high yield (Seenaiah *et al.*, 2014 ▸). We have recently reported the synthesis of a variety of anti­metabolites starting from activated and unsaturated nitriles (Abu-Zaied *et al.*, 2024 ▸; Mohamed-Ezzat & Elgemeie, 2024 ▸). The reaction between 2-(benzo[*d*]thia­zol-2-yl)-3-phenyl­acryl­amide, **1**, and ethyl cyano­acetate in refluxing ethanol containing a small amount of piperidine (initially intended as a catalyst) was examined as part of this program (Fig. 1 ▸). The product was shown to be neither of the expected condensed benzo­thia­zolo[3,2-*a*]pyridines (**8** or **9**) but rather the 1:1 adduct **10** of (*E*)-5-(benzo[*d*]thia­zol-2(3*H*)-yl­idene)-1,2,5,6-tetra­hydro-2,6-dioxo-4-phenyl­pyridine-3-carbo­nitrile with its piperidinium salt. We assume that the formation of **10** proceeds *via* addition of the active methyl­ene group of ethyl cyano­acetate to the double bond of **1**, followed by cyclization *via* elimination of EtOH to give the inter­mediate **2**. This is then oxidized under the reaction conditions, formally losing one mol­ecule of hydrogen to give the inter­mediate **4** or its tautomer **7**. The latter clearly forms a pyridinium salt under the reaction conditions, and this in turn forms the 1:1 adduct **10** on crystallization. The chemical structure of **10** is consistent with elemental analysis and spectroscopic data and was determined unambiguously by single-crystal X-ray diffraction structural analysis.
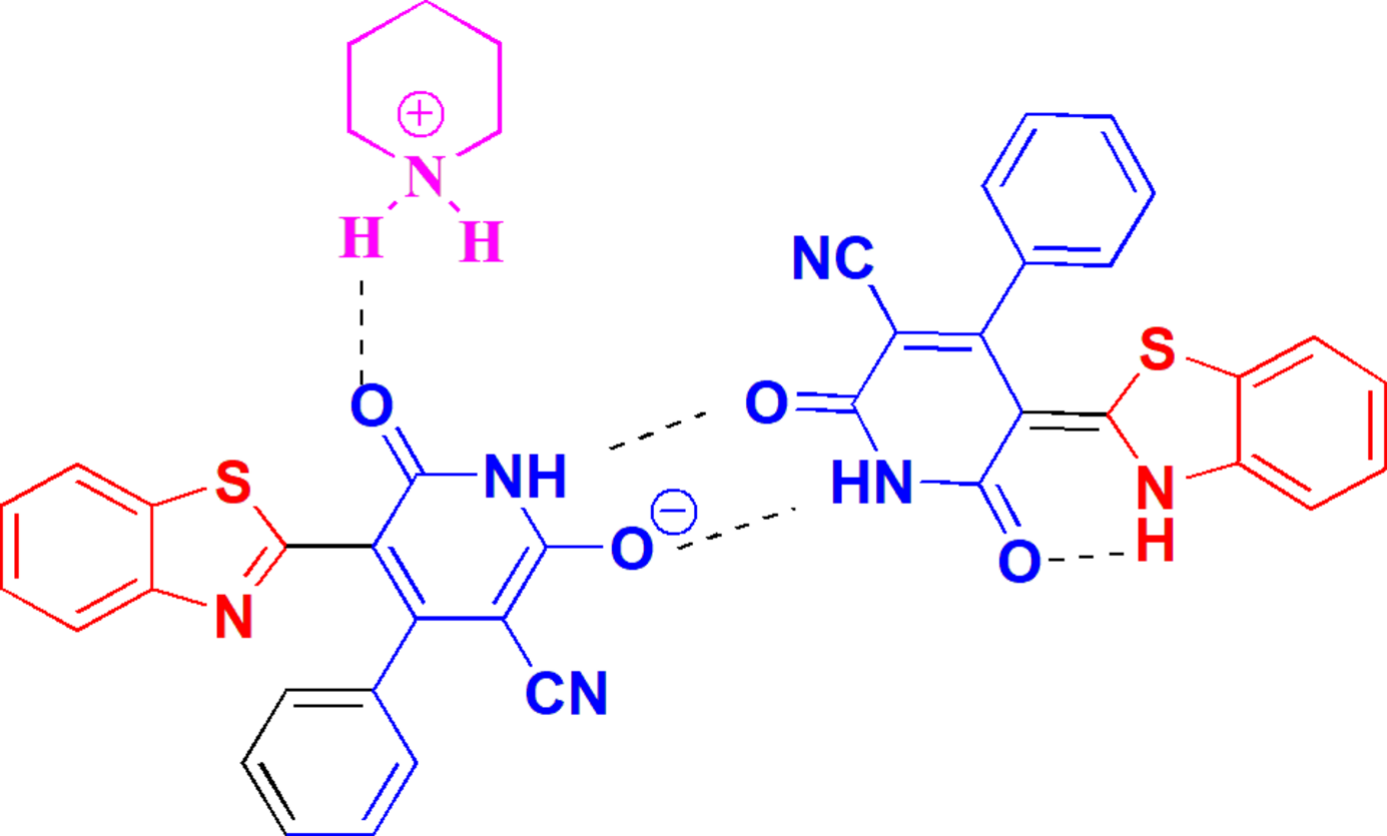


## 2. Structural commentary

**Figure 2:**
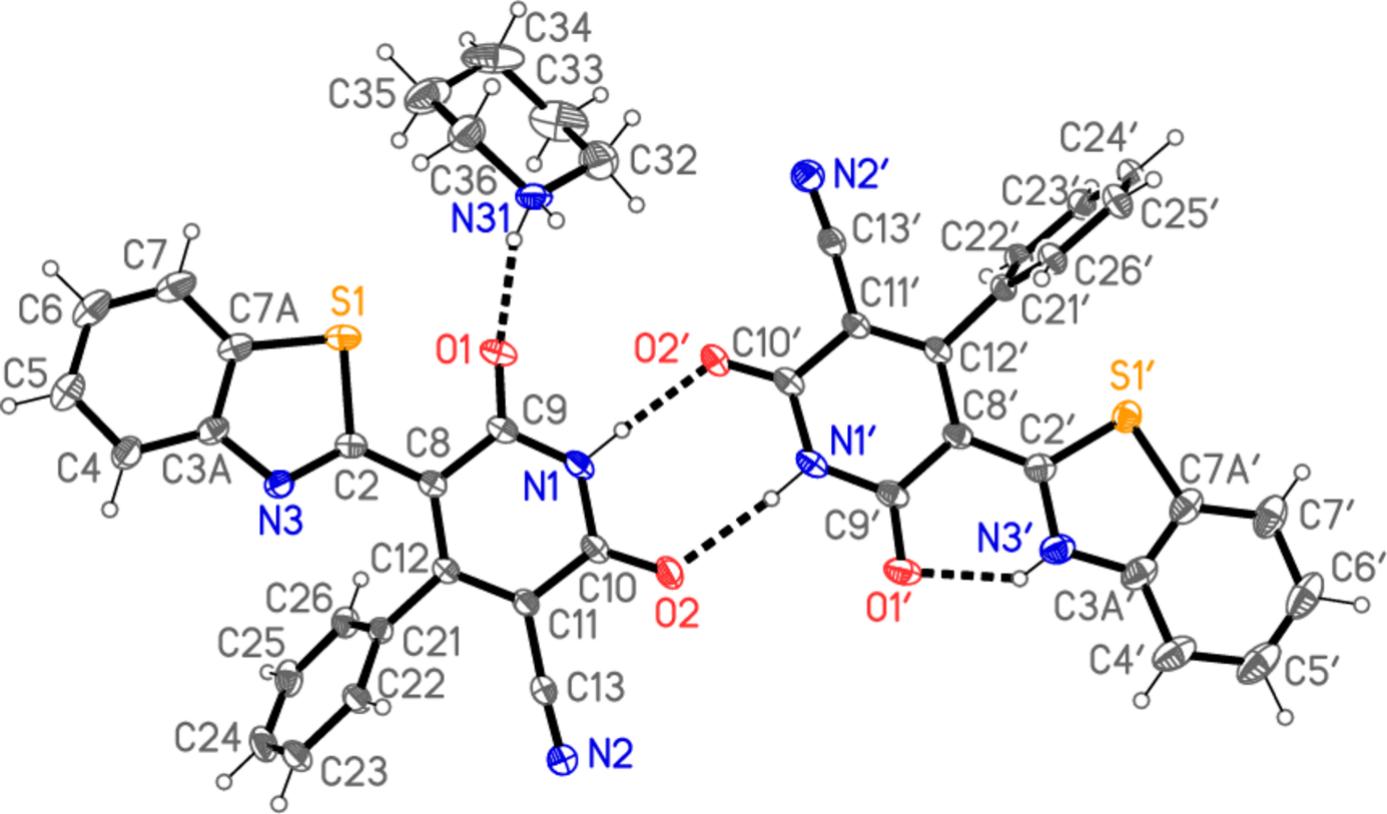
The formula unit of adduct **10** in the crystal. Ellipsoids represent 50% probability levels. Dashed lines indicate hydrogen bonds within the asymmetric unit.

The structure of the adduct **10** is shown in Fig. 2 ▸; hydrogen bonds between residues are discussed in *Supra­molecular features*. The anion and the neutral mol­ecule of **7** were assigned the same atom numbering (which corresponds to standard numbering for the benzo­thia­zole moieties), but the latter has atom names with primes (′). Table 1 ▸ presents a selection of paired mol­ecular dimensions, with values in the left column for the anion and the corresponding values in the right column for the neutral mol­ecule.

**Figure 3:**
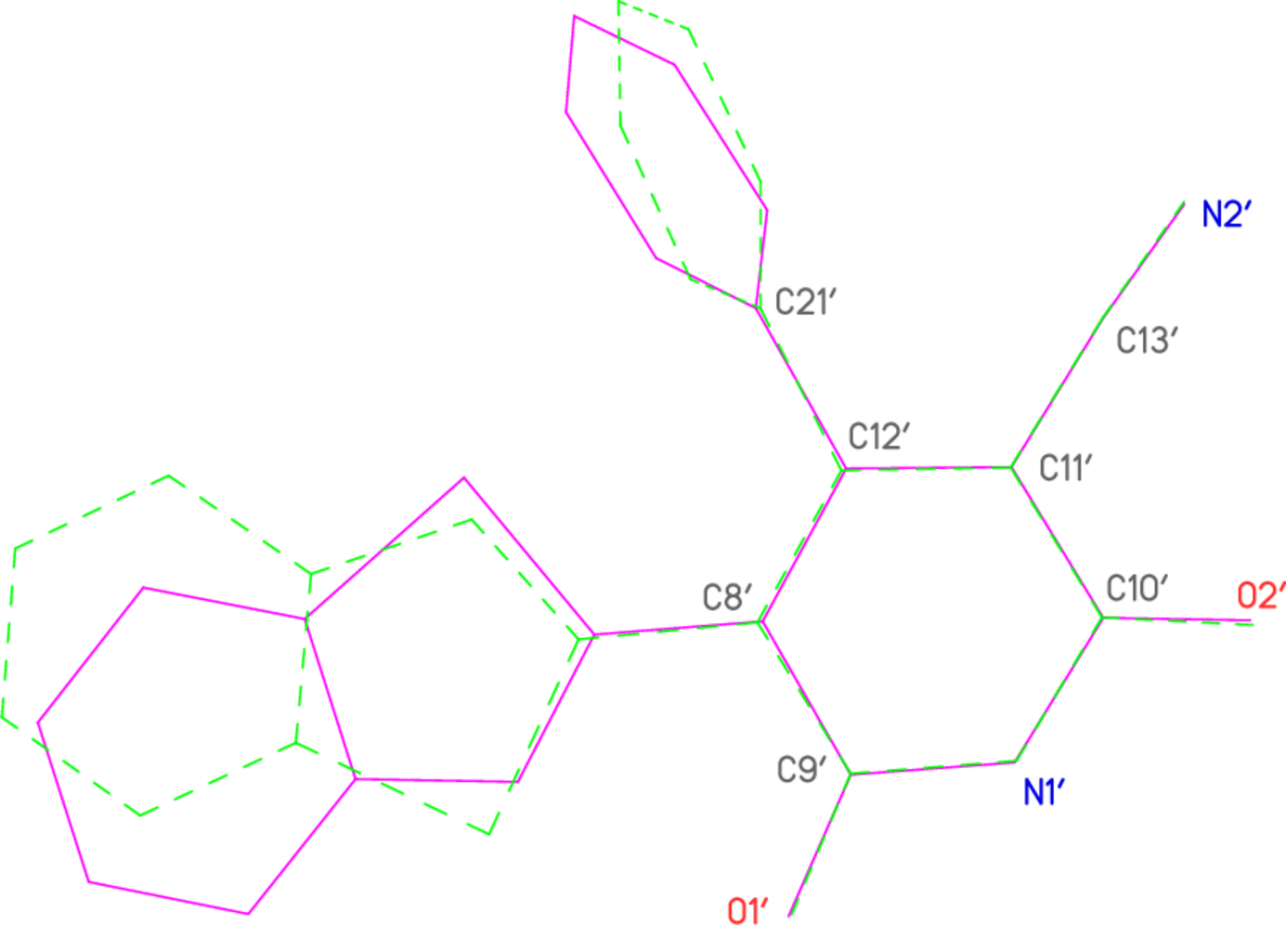
Least-squares fit of the neutral mol­ecule and anion of **10**. The former is drawn purple and the latter green. Fitted atoms are labelled. The r.m.s. deviation is 0.07 Å.

In the neutral mol­ecule, the heterocyclic nitro­gen atom N3′ is protonated. The two main residues display different configurations about the bonds C2—C8/C2′—C8′ between the approximately coplanar benzo­thia­zole and pyridinic ring systems, with an *E* configuration for the neutral mol­ecule, facilitating the intra­molecular hydrogen bond N3′—H⋯O1′, but a *Z* configuration for the anion, allowing a short intra­molecular S1⋯O1 contact of 2.5794 (10) Å. We have observed several such S⋯O contacts in related heterocyclic systems, *e.g.* 2.5992 (4) Å in 1-amino-3-(4-chloro­phen­yl)-2-cyano-3*H*-benzo[4,5]thia­zolo[3,2-*a*]pyridine-4-carboxamide (Metwally *et al.*, 2025 ▸). Torsion angles about the C2—C8 bonds are given in Table 1 ▸. A least-squares fit of the pyridinic rings of both residues makes the difference clear (Fig. 3 ▸). The inter­planar angles to the central pyridinic ring are: for the anion, phenyl 57.30 (3)° and benzo­thia­zole 15.34 (5)° and for the neutral mol­ecule, phenyl 79.01 (4)° and benzo­thia­zole 6.00 (5)°.

The resonance formulae given in the scheme are clearly an oversimplification, since extensive delocalization of formal double bonds can be expected, especially for the anion. For example, the formal negative charge at O2 of the anion is not reflected in any major differences in the four C—O bond lengths (including those of the neutral mol­ecule), which lie in the range 1.2365 (13)–1.2591 (13) Å, corresponding to delocalized double-bond character [the ‘standard’ table of bond lengths (Allen *et al.*, 1987 ▸) gives C—O bond lengths of 1.192 (5) Å for aldehydes, 1.210 (8) Å for ketones and 1.254 (10) Å for carboxyl­ates; a more recent (2023) anonymous inter­net summary gives 1.22 (2) Å for aldehydes and ketones, grouped together, and 1.25 (2) Å for carboxyl­ates (https://www.chem.uzh.ch/en/research/services/xray/bond_lenghts.html (*sic*)]. The formal double bonds C2′—C8′ and C2—N3 are significantly shorter than their formally single bond counterparts C2—C8 and C2′—N3′; the shorter C2—N3 bond is compensated for in the five-membered ring by the longer S1—C2 bond. Two of the angles in the five-membered rings differ appreciably; at N3/N3′ the angle is some 4.5° narrower for the anion, and the angle at C2/C2′ is correspondingly wider for the anion, preserving the angle sum of 540°. The exocyclic angles at C2/C2′ also differ notably; particularly striking is the very wide angle of 128.07 (8)° at C2′ of the neutral mol­ecule, which may perhaps be attributed to the close 1,5 approach of S1′ to the phenyl ring, with S1′⋯C21′ = 2.9190 (10) Å. The angle sums at C2 and C2′ are 359.97°.

## 3. Supra­molecular features

**Figure 4:**
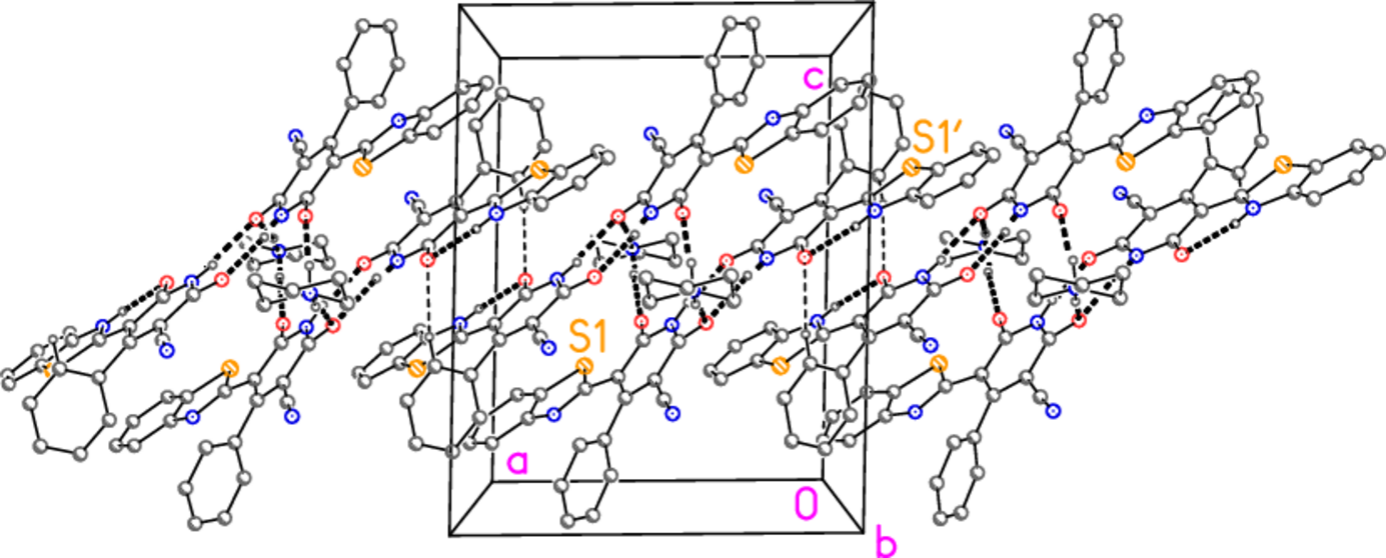
The packing of compound **10** viewed parallel to the *b* axis. Hydrogen atoms not involved in hydrogen bonding are omitted for clarity. Dashed lines indicate classical (thick) or ‘weak’ (thin) hydrogen bonds. Atom labels indicate the asymmetric unit.

Hydrogen bonds are listed in Table 2 ▸. Within the asymmetric unit (Fig. 2 ▸), the piperidinium cation is hydrogen bonded *via* H031 to atom O1 of the anion; the anion and the neutral mol­ecule are connected by the hydrogen bonds H01⋯O2′ and H01′⋯O2, which together form a ring of the well-known graph set 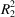
(8). Asymmetric units are then connected to form inversion-symmetric dimers by the hydrogen bond H032⋯O2(1 − *x*, 1 − *y*, 1 − *z*). These dimers are further linked by the ‘weak’ but very short hydrogen bond H22′⋯O1′(−*x*, 1 − *y*, 1 − *z*), connecting adjacent neutral mol­ecules, to form a broad ribbon of residues parallel to the *a* axis (Fig. 4 ▸). Three further C—H⋯O hydrogen bonds, within the dimeric units, are not shown in Fig. 4 ▸ but are given in Table 2 ▸.

## 4. Database survey

The searches employed Version 2024.3.0 of the routine ConQuest (Bruno *et al.*, 2002 ▸), as contained in the Cambridge Structural Database (Groom *et al.*, 2016 ▸). A search for the 1,3-benzo­thia­zole framework with no substituents (other than H) at the benzo group, a hydrogen atom at N3 and a substituent at C2, two bonded atoms at sulfur and three at nitro­gen, gave 113 hits (organic ordered structures only). Restricting the search to a carbon atom substituent at C2 reduced the number of hits to 37. Restricting the number of bonded atoms at C2 to three (corresponding to an exocyclic double bond at C2) and rejecting metal-bearing and ionic species led to eight final hits. Curiously, two of the hits correspond to a duplicated structure, with two apparently different datasets but three common authors [(*E*)-4-(2,3-di­hydro-1,3-benzo­thia­zol-2-yl­idene)-3-methyl-1-phenyl-1*H*-pyrazol-5(4*H*)-one, refcodes NUQBIL and NUQBIL01, Chakibe *et al.* (2010 ▸) and Chakib *et al.* (2019 ▸)]. Furthermore, the structure of 6-[3-(2-benzo­thia­zol­yl)pyridin-2-yl­thio]-*N*-[3-(2-benzo­thia­zol­yl)pyridin-2-yl]aniline (QEKNIE; De Souza *et al.*, 2006 ▸) has what seems to be an erroneously placed hydrogen at one of the two N3 atoms; since no structure factors were deposited, this cannot be checked. The other hits were 1,3-benzo­thia­zol-2(3*H*)-ylidenemalonaldehyde [AYOMAN, Ennajih *et al.* (2011 ▸), with an intra­molecular S⋯O contact of 2.763 Å]; 3-[1,3-benzo­thia­zol-2(3*H*)-yl­idene]-4-(4-bromo­phen­yl)-2,4-dioxo-*N*-phenyl­butan­amide [DOQFAU, Lystsova *et al.* (2024 ▸), S⋯O 2.672 Å]; 2-(3*H*-benzo­thia­zol-2-yl­idene)-2-cyano­thio­acetamide [GIYZIY, Basheer & Rappoport (2008 ▸)]; 2-(1,3-benzo­thia­zol-2(3*H*)-yl­idene)cyclo­hexane-1,3-dione [SOTHUH, Kumar & Ila (2019 ▸), S⋯O 2.646 Å]; and 2-[1,3-benzo­thia­zol-2(3*H*)-yl­idene]-5,7-di-*t*-butyl-4-nitro­cyclo­hepta-4,6-diene-1,3-dione [TADXIJ, Tkachev (2020 ▸), S⋯O 2.564 Å].

## 5. Synthesis and crystallization

A mixture of 2-(benzo[*d*]thia­zol-2-yl)-3-phenyl­acryl­amide (0.01 mol, 2.68 g), and ethyl 2-cyano­acetate (0.01 mole, 1.13 g) was dissolved in 70 mL of ethanol, and *ca*. 0.085 g (0.01 mmol) piperidine were added. The reaction mixture was stirred under reflux for 5 h. After cooling, the buff-coloured crystals thus obtained were filtered, washed with ethanol and dried at room temperature. Yield: 87%, m.p.: > 573 K. IR (KBr): ν (cm^−1^) = 3240 (NH), 2958 (CH, aromatic), 2220 (CN), 1674 (C=O); ^1^H NMR (400 MHz, DMSO-*d*_6_): *δ_H_* 1.49 (*d*, 2H, *J* = 4.76 Hz, piperidine-H), 1.62 (*d*, 4H, *J* = 5.04 Hz, piperidine-H), 3.00 (*t*, 4H, *J* = 5.76 Hz, piperidine-H), 7.33–7.36 (*m*, 2H, Ar-H), 7.43–7.52 (*m*, 6H, Ar-H), 7.62–7.70 (*m*, 6H, Ar-H), 7.82 (*d*, 2H, *J* = 7.96 Hz, Ar-H), 8.03 (*d*, 2H, *J* = 8.16 Hz, Ar-H), 11.68 (*br*, 1H, NH) ppm; ^13^C NMR (100 MHz, DMSO-*d*_6_): *δ_C_* = 22.09, 22.71, 44.29, 83.12, 102.20, 120.36, 121.13, 121.27, 123.26, 125.15, 128.24, 128.43, 135.06, 136.36, 137.47, 152.22, 158.79, 163.73, 163.88 ppm. Analysis: Calculated for C_43_H_33_N_7_O_4_S_2_ (775.88): C 66.56, H 4.29, N 12.64, S 8.26%. Found: C 66.76, H 4.36, N 12.44, S 8.40%.

## 6. Refinement

Details of data collection and structure refinement are summarized in Table 3 ▸. The hydrogen atoms of the NH groups were refined freely. Other hydrogen atoms were included using a riding model starting from calculated positions (C—H_methyl­ene_ = 0.99, C—H_arom_ = 0.95 Å). The *U*(H) values were fixed at 1.2 × *U*_eq_ of the parent carbon atoms.

## Supplementary Material

Crystal structure: contains datablock(s) I, global. DOI: 10.1107/S2056989025006991/yz2070sup1.cif

Structure factors: contains datablock(s) I. DOI: 10.1107/S2056989025006991/yz2070Isup3.hkl

CCDC reference: 2478343

Additional supporting information:  crystallographic information; 3D view; checkCIF report

## Figures and Tables

**Table 1:** Selected geometric parameters (Å, °)

S1—C2	1.7742 (10)	S1′—C2′	1.7331 (11)
S1—C7*A*	1.7265 (13)	S1′—C7*A*′	1.7422 (12)
C2—N3	1.3058 (13)	C2′—N3′	1.3417 (14)
C2—C8	1.4610 (14)	C2′—C8′	1.4317 (15)
N3—C3*A*	1.3828 (13)	N3′—C3*A*′	1.3830 (16)
C3*A*—C7*A*	1.4033 (15)	C3*A*′—C7*A*′	1.3898 (19)
C9—O1	1.2446 (13)	C9′—O1′	1.2523 (13)
C10—O2	1.2591 (13)	C10′—O2′	1.2365 (13)
			
C7*A*—S1—C2	89.05 (5)	C2′—S1′—C7*A*′	91.12 (6)
N3—C2—C8	124.75 (9)	N3′—C2′—C8′	121.35 (10)
N3—C2—S1	114.49 (8)	N3′—C2′—S1′	110.55 (8)
C8—C2—S1	120.73 (7)	C8′—C2′—S1′	128.07 (8)
C2—N3—C3*A*	111.53 (9)	C2′—N3′—C3*A*′	116.07 (11)
N3—C3*A*—C7*A*	114.97 (10)	N3′—C3*A*′—C7*A*′	111.40 (10)
C3*A*—C7*A*—S1	109.95 (8)	C3*A*′—C7*A*′—S1′	110.84 (9)
C10—N1—C9	126.18 (9)	C9′—N1′—C10′	126.23 (9)
			
N3—C2—C8—C12	11.74 (15)	N3′—C2′—C8′—C12′	175.67 (9)
S1—C2—C8—C12	−170.52 (7)	S1′—C2′—C8′—C12′	−6.52 (15)
N3—C2—C8—C9	−164.55 (9)	N3′—C2′—C8′—C9′	−4.04 (14)
S1—C2—C8—C9	13.19 (12)	S1′—C2′—C8′—C9′	173.77 (7)

**Table 2:** Hydrogen-bond geometry (Å, °)

*D*—H⋯*A*	*D*—H	H⋯*A*	*D*⋯*A*	*D*—H⋯*A*
N1—H01⋯O2′	0.888 (18)	1.887 (18)	2.7703 (11)	172.9 (16)
N3′—H03′⋯O1′	0.804 (19)	1.878 (19)	2.5399 (15)	139.0 (18)
N1′—H01′⋯O2	0.883 (17)	2.016 (18)	2.8903 (11)	169.8 (16)
N31—H031⋯O1	0.86 (2)	1.91 (2)	2.7327 (12)	158.7 (18)
N31—H032⋯O2^i^	0.87 (2)	1.88 (2)	2.7322 (13)	169.3 (19)
C36—H36*B*⋯O1′^i^	0.99	2.52	3.4487 (16)	157
C22′—H22′⋯O1′^ii^	0.95	2.34	3.2691 (13)	165
C26′—H26′⋯N3^i^	0.95	2.44	3.3437 (13)	159

Symmetry codes: (i) 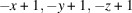; (ii) 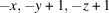.

**Table 3:** Experimental details

Crystal data	
Chemical formula	C_5_H_12_N^+^·C_19_H_10_N_3_O_2_S^−^·C_19_H_11_N_3_O_2_S
*M*_r_	775.88
Crystal system, space group	Monoclinic, *P*2_1_/*c*
Temperature (K)	100
*a*, *b*, *c* (Å)	11.3927 (2), 22.2956 (5), 14.6155 (3)
β (°)	91.4989 (18)
*V* (Å^3^)	3711.15 (13)
*Z*	4
Radiation type	Mo *K*α
μ (mm^−1^)	0.20
Crystal size (mm)	0.20 × 0.18 × 0.12

Data collection	
Diffractometer	XtaLAB Synergy
Absorption correction	Multi-scan (*CrysAlis PRO*; Rigaku OD, 2024 ▸)
*T*_min_, *T*_max_	0.833, 1.000
No. of measured, independent and observed [*I* > 2σ(*I*)] reflections	308721, 17991, 13421
*R*_int_	0.058
θ values (°)	θ_max_ = 36.3, θ_min_ = 2.0
(sin θ/λ)_max_ (Å^−1^)	0.833

Refinement	
*R*[*F*^2^ > 2σ(*F*^2^)], *wR*(*F*^2^), *S*	0.048, 0.130, 1.01
No. of reflections	17991
No. of parameters	525
H-atom treatment	H atoms treated by a mixture of independent and constrained refinement
Δρ_max_, Δρ_min_ (e Å^−3^)	0.54, −0.43

Computer programs: *CrysAlis PRO* (Rigaku OD, 2024 ▸), *SHELXT* (Sheldrick, 2015*a* ▸), *SHELXL2019/3* (Sheldrick, 2015*b* ▸), *XP* (Bruker, 1998 ▸) and *publCIF* (Westrip, 2010 ▸).

## supplementary crystallographic information

### Piperidinium (Z)-5-(benzo[d]thiazol-2-yl)-3-cyano-6-oxo-4-phenyl-1,6-dihydropyridin-2-olate–(E)-5-(2,3-dihydrobenzo[d]thiazol-2-ylidene)-2,6-dioxo-4-phenyl-1,2,5,6-tetrahydropyridine-3-carbonitrile (1/1) Crystal data

**Table:**

C_5_H_12_N^+^·C_19_H_10_N_3_O_2_S^−^·C_19_H_11_N_3_O_2_S·C_5_H_12_N	*F*(000) = 1616
*M**_r_* = 775.88	*D*_x_ = 1.389 Mg m^−^^3^
Monoclinic, *P*2_1_/*c*	Mo *K*α radiation, λ = 0.71073 Å
*a* = 11.3927 (2) Å	Cell parameters from 99329 reflections
*b* = 22.2956 (5) Å	θ = 2.3–41.4°
*c* = 14.6155 (3) Å	µ = 0.20 mm^−^^1^
β = 91.4989 (18)°	*T* = 100 K
*V* = 3711.15 (13) Å^3^	Block, pale orange
*Z* = 4	0.20 × 0.18 × 0.12 mm

### Piperidinium (Z)-5-(benzo[d]thiazol-2-yl)-3-cyano-6-oxo-4-phenyl-1,6-dihydropyridin-2-olate–(E)-5-(2,3-dihydrobenzo[d]thiazol-2-ylidene)-2,6-dioxo-4-phenyl-1,2,5,6-tetrahydropyridine-3-carbonitrile (1/1) Data collection

**Table:**

XtaLAB Synergy diffractometer	17991 independent reflections
Radiation source: micro-focus sealed X-ray tube, PhotonJet (Mo) X-ray Source	13421 reflections with *I* > 2σ(*I*)
Mirror monochromator	*R*_int_ = 0.058
Detector resolution: 10.0000 pixels mm^-1^	θ_max_ = 36.3°, θ_min_ = 2.0°
ω scans	*h* = −18→18
Absorption correction: multi-scan (CrysAlisPro; Rigaku OD, 2024)	*k* = −37→37
*T*_min_ = 0.833, *T*_max_ = 1.000	*l* = −24→24
308721 measured reflections	

### Piperidinium (Z)-5-(benzo[d]thiazol-2-yl)-3-cyano-6-oxo-4-phenyl-1,6-dihydropyridin-2-olate–(E)-5-(2,3-dihydrobenzo[d]thiazol-2-ylidene)-2,6-dioxo-4-phenyl-1,2,5,6-tetrahydropyridine-3-carbonitrile (1/1) Refinement

**Table:**

Refinement on *F*^2^	Primary atom site location: dual
Least-squares matrix: full	Hydrogen site location: mixed
*R*[*F*^2^ > 2σ(*F*^2^)] = 0.048	H atoms treated by a mixture of independent and constrained refinement
*wR*(*F*^2^) = 0.130	*w* = 1/[σ^2^(*F*_o_^2^) + (0.0621*P*)^2^ + 1.247*P*] where *P* = (*F*_o_^2^ + 2*F*_c_^2^)/3
*S* = 1.01	(Δ/σ)_max_ = 0.001
17991 reflections	Δρ_max_ = 0.54 e Å^−^^3^
525 parameters	Δρ_min_ = −0.43 e Å^−^^3^
0 restraints	

### Piperidinium (Z)-5-(benzo[d]thiazol-2-yl)-3-cyano-6-oxo-4-phenyl-1,6-dihydropyridin-2-olate–(E)-5-(2,3-dihydrobenzo[d]thiazol-2-ylidene)-2,6-dioxo-4-phenyl-1,2,5,6-tetrahydropyridine-3-carbonitrile (1/1) Fractional atomic coordinates and isotropic or equivalent isotropic displacement parameters (Å^2^)

**Table:**

	*x*	*y*	*z*	*U*_iso_*/*U*_eq_	
S1	0.71164 (3)	0.33569 (2)	0.28714 (2)	0.02558 (6)	
C2	0.70539 (9)	0.40954 (4)	0.24208 (7)	0.01945 (16)	
N3	0.79003 (7)	0.42271 (4)	0.18698 (6)	0.02095 (15)	
C3A	0.86519 (9)	0.37470 (5)	0.17600 (7)	0.02228 (17)	
C4	0.96547 (10)	0.37498 (5)	0.12237 (8)	0.0280 (2)	
H4	0.986670	0.409828	0.089244	0.034*	
C5	1.03307 (11)	0.32333 (6)	0.11867 (10)	0.0342 (3)	
H5	1.100731	0.322773	0.082038	0.041*	
C6	1.00311 (12)	0.27198 (6)	0.16813 (11)	0.0372 (3)	
H6	1.050738	0.237131	0.164531	0.045*	
C7	0.90580 (12)	0.27128 (5)	0.22178 (10)	0.0342 (3)	
H7	0.886269	0.236544	0.255821	0.041*	
C7A	0.83611 (10)	0.32294 (5)	0.22512 (8)	0.02545 (19)	
C8	0.61355 (8)	0.45117 (4)	0.26917 (6)	0.01869 (15)	
C9	0.54643 (9)	0.43430 (5)	0.34723 (7)	0.02114 (17)	
C10	0.44963 (9)	0.53224 (5)	0.34739 (7)	0.02072 (16)	
C11	0.51217 (9)	0.54763 (4)	0.26706 (6)	0.01932 (16)	
C12	0.59153 (8)	0.50714 (4)	0.22774 (6)	0.01743 (15)	
C13	0.48682 (9)	0.60528 (5)	0.22925 (7)	0.02280 (18)	
N1	0.46867 (8)	0.47575 (4)	0.38062 (6)	0.02260 (16)	
H01	0.4263 (15)	0.4639 (8)	0.4275 (12)	0.037 (4)*	
N2	0.46324 (9)	0.65278 (5)	0.20303 (8)	0.0316 (2)	
O1	0.55326 (8)	0.38457 (4)	0.38568 (6)	0.02875 (16)	
O2	0.37784 (7)	0.56595 (4)	0.38654 (5)	0.02544 (15)	
C21	0.64521 (8)	0.52531 (4)	0.14002 (6)	0.01792 (15)	
C22	0.70857 (9)	0.57856 (5)	0.13397 (7)	0.02216 (17)	
H22	0.719625	0.603117	0.186611	0.027*	
C23	0.75581 (10)	0.59597 (6)	0.05105 (8)	0.0283 (2)	
H23	0.800085	0.631968	0.047631	0.034*	
C24	0.73836 (10)	0.56089 (6)	−0.02646 (8)	0.0307 (2)	
H24	0.770665	0.572789	−0.082918	0.037*	
C25	0.67349 (10)	0.50831 (6)	−0.02130 (7)	0.0286 (2)	
H25	0.660566	0.484542	−0.074554	0.034*	
C26	0.62730 (9)	0.49028 (5)	0.06161 (7)	0.02262 (17)	
H26	0.583547	0.454099	0.064855	0.027*	
S1'	−0.16760 (2)	0.54952 (2)	0.71007 (2)	0.02374 (6)	
C2'	−0.05035 (9)	0.57006 (5)	0.64368 (7)	0.02178 (17)	
N3'	−0.06080 (10)	0.62743 (4)	0.61697 (7)	0.02728 (18)	
H03'	−0.0104 (16)	0.6404 (8)	0.5850 (13)	0.041 (5)*	
C3A'	−0.15731 (12)	0.65807 (5)	0.64839 (8)	0.0296 (2)	
C4'	−0.18492 (14)	0.71833 (6)	0.63328 (10)	0.0385 (3)	
H4'	−0.137277	0.743219	0.596763	0.046*	
C5'	−0.28451 (15)	0.74025 (6)	0.67371 (10)	0.0435 (4)	
H5'	−0.305249	0.781198	0.665437	0.052*	
C6'	−0.35485 (14)	0.70369 (7)	0.72613 (10)	0.0425 (3)	
H6'	−0.423517	0.720059	0.751898	0.051*	
C7'	−0.32756 (12)	0.64384 (6)	0.74194 (9)	0.0350 (3)	
H7'	−0.375404	0.619173	0.778637	0.042*	
C7A'	−0.22680 (11)	0.62141 (5)	0.70160 (8)	0.0282 (2)	
C8'	0.04555 (9)	0.53358 (4)	0.61541 (6)	0.01990 (16)	
C9'	0.12669 (10)	0.55998 (5)	0.55169 (7)	0.02201 (17)	
C10'	0.23707 (9)	0.46552 (5)	0.54682 (7)	0.02103 (17)	
C11'	0.15670 (8)	0.44088 (4)	0.61192 (6)	0.01916 (16)	
C12'	0.06532 (8)	0.47404 (4)	0.64627 (6)	0.01820 (15)	
C13'	0.18219 (9)	0.38101 (5)	0.64011 (7)	0.02272 (18)	
N1'	0.21530 (8)	0.52401 (4)	0.52096 (6)	0.02232 (16)	
H01'	0.2676 (15)	0.5403 (8)	0.4849 (12)	0.034 (4)*	
N2'	0.20876 (9)	0.33293 (5)	0.65995 (8)	0.0329 (2)	
O1'	0.12078 (8)	0.61269 (4)	0.52232 (6)	0.02846 (16)	
O2'	0.32082 (7)	0.43730 (4)	0.51621 (6)	0.02727 (16)	
C21'	−0.00711 (8)	0.44752 (4)	0.71946 (6)	0.01795 (15)	
C22'	−0.09844 (9)	0.40759 (5)	0.69898 (7)	0.02164 (17)	
H22'	−0.113584	0.395214	0.637603	0.026*	
C23'	−0.16728 (9)	0.38600 (5)	0.76928 (8)	0.02446 (19)	
H23'	−0.230441	0.359392	0.755511	0.029*	
C24'	−0.14416 (10)	0.40310 (5)	0.85906 (8)	0.0269 (2)	
H24'	−0.191538	0.388380	0.906654	0.032*	
C25'	−0.05148 (10)	0.44184 (5)	0.87938 (7)	0.0266 (2)	
H25'	−0.035010	0.453075	0.941086	0.032*	
C26'	0.01712 (9)	0.46420 (5)	0.81015 (7)	0.02136 (17)	
H26'	0.080264	0.490733	0.824330	0.026*	
N31	0.58360 (10)	0.32155 (4)	0.54491 (7)	0.02745 (18)	
H031	0.5776 (17)	0.3327 (9)	0.4883 (14)	0.047 (5)*	
H032	0.5914 (17)	0.3556 (9)	0.5729 (13)	0.046 (5)*	
C32	0.47111 (13)	0.29215 (7)	0.56629 (12)	0.0422 (3)	
H32A	0.404921	0.318311	0.546616	0.051*	
H32B	0.466803	0.286055	0.633215	0.051*	
C33	0.46082 (18)	0.23233 (8)	0.51799 (13)	0.0558 (5)	
H33A	0.452938	0.239223	0.451199	0.067*	
H33B	0.388859	0.211715	0.537840	0.067*	
C34	0.56620 (19)	0.19212 (6)	0.53749 (10)	0.0535 (5)	
H34A	0.568542	0.180584	0.602922	0.064*	
H34B	0.558939	0.155062	0.500446	0.064*	
C35	0.67791 (18)	0.22428 (7)	0.51472 (11)	0.0488 (4)	
H35A	0.679198	0.231704	0.447972	0.059*	
H35B	0.746045	0.198617	0.531503	0.059*	
C36	0.68824 (12)	0.28326 (6)	0.56510 (10)	0.0350 (3)	
H36A	0.695068	0.275690	0.631769	0.042*	
H36B	0.760063	0.304450	0.546279	0.042*	

### Piperidinium (Z)-5-(benzo[d]thiazol-2-yl)-3-cyano-6-oxo-4-phenyl-1,6-dihydropyridin-2-olate–(E)-5-(2,3-dihydrobenzo[d]thiazol-2-ylidene)-2,6-dioxo-4-phenyl-1,2,5,6-tetrahydropyridine-3-carbonitrile (1/1) Atomic displacement parameters (Å^2^)

**Table:**

	*U*^11^	*U*^22^	*U*^33^	*U*^12^	*U*^13^	*U*^23^
S1	0.03005 (13)	0.01907 (11)	0.02744 (12)	−0.00193 (9)	−0.00306 (9)	0.00599 (9)
C2	0.0217 (4)	0.0177 (4)	0.0187 (4)	−0.0024 (3)	−0.0035 (3)	0.0013 (3)
N3	0.0206 (3)	0.0190 (3)	0.0232 (4)	0.0008 (3)	−0.0006 (3)	0.0011 (3)
C3A	0.0224 (4)	0.0197 (4)	0.0245 (4)	0.0018 (3)	−0.0047 (3)	−0.0004 (3)
C4	0.0247 (5)	0.0268 (5)	0.0326 (5)	0.0053 (4)	0.0006 (4)	−0.0001 (4)
C5	0.0285 (5)	0.0320 (6)	0.0418 (7)	0.0092 (4)	−0.0020 (5)	−0.0045 (5)
C6	0.0340 (6)	0.0262 (5)	0.0510 (8)	0.0101 (4)	−0.0082 (5)	−0.0045 (5)
C7	0.0358 (6)	0.0201 (5)	0.0463 (7)	0.0039 (4)	−0.0089 (5)	0.0031 (4)
C7A	0.0273 (5)	0.0190 (4)	0.0297 (5)	0.0006 (3)	−0.0070 (4)	0.0014 (4)
C8	0.0205 (4)	0.0190 (4)	0.0166 (4)	−0.0026 (3)	0.0005 (3)	0.0014 (3)
C9	0.0236 (4)	0.0219 (4)	0.0180 (4)	−0.0047 (3)	0.0006 (3)	0.0026 (3)
C10	0.0223 (4)	0.0222 (4)	0.0179 (4)	−0.0050 (3)	0.0043 (3)	0.0002 (3)
C11	0.0207 (4)	0.0195 (4)	0.0180 (4)	−0.0015 (3)	0.0044 (3)	0.0014 (3)
C12	0.0178 (4)	0.0190 (4)	0.0155 (3)	−0.0025 (3)	0.0008 (3)	0.0006 (3)
C13	0.0214 (4)	0.0244 (4)	0.0229 (4)	0.0000 (3)	0.0073 (3)	0.0019 (3)
N1	0.0266 (4)	0.0230 (4)	0.0185 (3)	−0.0051 (3)	0.0061 (3)	0.0026 (3)
N2	0.0296 (5)	0.0274 (5)	0.0385 (5)	0.0048 (4)	0.0115 (4)	0.0076 (4)
O1	0.0355 (4)	0.0251 (4)	0.0257 (4)	−0.0031 (3)	0.0024 (3)	0.0096 (3)
O2	0.0269 (4)	0.0252 (4)	0.0247 (3)	−0.0039 (3)	0.0105 (3)	−0.0011 (3)
C21	0.0174 (4)	0.0203 (4)	0.0162 (3)	0.0018 (3)	0.0015 (3)	0.0021 (3)
C22	0.0217 (4)	0.0241 (4)	0.0209 (4)	−0.0019 (3)	0.0040 (3)	0.0029 (3)
C23	0.0254 (5)	0.0328 (5)	0.0272 (5)	−0.0011 (4)	0.0075 (4)	0.0095 (4)
C24	0.0260 (5)	0.0460 (7)	0.0204 (4)	0.0054 (4)	0.0073 (4)	0.0084 (4)
C25	0.0257 (5)	0.0427 (6)	0.0175 (4)	0.0073 (4)	0.0019 (3)	−0.0010 (4)
C26	0.0220 (4)	0.0271 (5)	0.0187 (4)	0.0026 (3)	0.0001 (3)	−0.0014 (3)
S1'	0.02431 (11)	0.02488 (12)	0.02196 (11)	0.00288 (9)	−0.00049 (8)	−0.00297 (9)
C2'	0.0280 (4)	0.0203 (4)	0.0169 (4)	−0.0007 (3)	−0.0033 (3)	−0.0015 (3)
N3'	0.0374 (5)	0.0212 (4)	0.0230 (4)	0.0016 (3)	−0.0023 (4)	−0.0004 (3)
C3A'	0.0399 (6)	0.0234 (5)	0.0250 (5)	0.0060 (4)	−0.0090 (4)	−0.0059 (4)
C4'	0.0550 (8)	0.0239 (5)	0.0358 (6)	0.0088 (5)	−0.0131 (6)	−0.0050 (4)
C5'	0.0582 (9)	0.0305 (6)	0.0407 (7)	0.0166 (6)	−0.0191 (6)	−0.0143 (5)
C6'	0.0459 (7)	0.0410 (7)	0.0398 (7)	0.0193 (6)	−0.0138 (6)	−0.0191 (6)
C7'	0.0337 (6)	0.0386 (6)	0.0322 (6)	0.0102 (5)	−0.0082 (5)	−0.0134 (5)
C7A'	0.0324 (5)	0.0274 (5)	0.0243 (5)	0.0071 (4)	−0.0085 (4)	−0.0083 (4)
C8'	0.0250 (4)	0.0191 (4)	0.0156 (4)	−0.0030 (3)	0.0001 (3)	0.0008 (3)
C9'	0.0299 (5)	0.0207 (4)	0.0154 (4)	−0.0070 (3)	−0.0006 (3)	0.0007 (3)
C10'	0.0220 (4)	0.0239 (4)	0.0172 (4)	−0.0058 (3)	0.0019 (3)	0.0025 (3)
C11'	0.0196 (4)	0.0198 (4)	0.0183 (4)	−0.0037 (3)	0.0029 (3)	0.0024 (3)
C12'	0.0199 (4)	0.0197 (4)	0.0150 (3)	−0.0041 (3)	0.0000 (3)	0.0010 (3)
C13'	0.0185 (4)	0.0252 (4)	0.0246 (4)	−0.0029 (3)	0.0052 (3)	0.0030 (3)
N1'	0.0256 (4)	0.0237 (4)	0.0179 (3)	−0.0072 (3)	0.0031 (3)	0.0036 (3)
N2'	0.0265 (4)	0.0287 (5)	0.0440 (6)	0.0001 (4)	0.0088 (4)	0.0094 (4)
O1'	0.0430 (5)	0.0201 (3)	0.0223 (3)	−0.0061 (3)	0.0019 (3)	0.0035 (3)
O2'	0.0251 (4)	0.0307 (4)	0.0265 (4)	−0.0019 (3)	0.0095 (3)	0.0058 (3)
C21'	0.0174 (4)	0.0191 (4)	0.0174 (4)	−0.0010 (3)	0.0022 (3)	0.0012 (3)
C22'	0.0214 (4)	0.0218 (4)	0.0218 (4)	−0.0036 (3)	0.0017 (3)	−0.0006 (3)
C23'	0.0211 (4)	0.0223 (4)	0.0302 (5)	−0.0034 (3)	0.0061 (3)	0.0006 (4)
C24'	0.0258 (5)	0.0285 (5)	0.0270 (5)	−0.0008 (4)	0.0106 (4)	0.0039 (4)
C25'	0.0283 (5)	0.0333 (5)	0.0183 (4)	−0.0012 (4)	0.0049 (3)	0.0007 (4)
C26'	0.0209 (4)	0.0258 (4)	0.0174 (4)	−0.0020 (3)	0.0008 (3)	−0.0002 (3)
N31	0.0410 (5)	0.0176 (4)	0.0238 (4)	0.0023 (3)	0.0030 (4)	0.0047 (3)
C32	0.0350 (6)	0.0430 (7)	0.0487 (8)	−0.0018 (5)	0.0039 (6)	0.0075 (6)
C33	0.0655 (11)	0.0483 (9)	0.0524 (9)	−0.0254 (8)	−0.0220 (8)	0.0077 (7)
C34	0.1079 (15)	0.0193 (5)	0.0322 (6)	−0.0086 (7)	−0.0215 (8)	0.0013 (5)
C35	0.0772 (12)	0.0340 (7)	0.0352 (7)	0.0242 (7)	−0.0022 (7)	−0.0041 (5)
C36	0.0344 (6)	0.0295 (6)	0.0411 (7)	0.0039 (4)	0.0012 (5)	−0.0005 (5)

### Piperidinium (Z)-5-(benzo[d]thiazol-2-yl)-3-cyano-6-oxo-4-phenyl-1,6-dihydropyridin-2-olate–(E)-5-(2,3-dihydrobenzo[d]thiazol-2-ylidene)-2,6-dioxo-4-phenyl-1,2,5,6-tetrahydropyridine-3-carbonitrile (1/1) Geometric parameters (Å, º)

**Table:**

S1—C2	1.7742 (10)	C12'—C21'	1.4902 (13)
S1—C7A	1.7265 (13)	C13'—N2'	1.1491 (15)
C2—N3	1.3058 (13)	C21'—C22'	1.3958 (13)
C2—C8	1.4610 (14)	C21'—C26'	1.3973 (14)
N3—C3A	1.3828 (13)	C22'—C23'	1.3946 (14)
C3A—C4	1.4023 (16)	C23'—C24'	1.3850 (16)
C3A—C7A	1.4033 (15)	C24'—C25'	1.3903 (17)
C4—C5	1.3872 (16)	C25'—C26'	1.3873 (14)
C5—C6	1.401 (2)	N31—C32	1.4801 (18)
C6—C7	1.375 (2)	N31—C36	1.4895 (17)
C7—C7A	1.4005 (16)	C32—C33	1.512 (2)
C8—C12	1.4067 (13)	C33—C34	1.519 (3)
C8—C9	1.4399 (13)	C34—C35	1.506 (3)
C9—O1	1.2446 (13)	C35—C36	1.510 (2)
C9—N1	1.3782 (14)	C4—H4	0.9500
C10—O2	1.2591 (13)	C5—H5	0.9500
C10—N1	1.3652 (14)	C6—H6	0.9500
C10—C11	1.4310 (13)	C7—H7	0.9500
C11—C12	1.4107 (13)	N1—H01	0.888 (18)
C11—C13	1.4257 (14)	C22—H22	0.9500
C12—C21	1.4910 (13)	C23—H23	0.9500
C13—N2	1.1553 (14)	C24—H24	0.9500
C21—C22	1.3934 (14)	C25—H25	0.9500
C21—C26	1.3975 (14)	C26—H26	0.9500
C22—C23	1.3940 (14)	N3'—H03'	0.804 (19)
C23—C24	1.3866 (18)	C4'—H4'	0.9500
C24—C25	1.3889 (19)	C5'—H5'	0.9500
C25—C26	1.3930 (15)	C6'—H6'	0.9500
S1'—C2'	1.7331 (11)	C7'—H7'	0.9500
S1'—C7A'	1.7422 (12)	N1'—H01'	0.883 (17)
C2'—N3'	1.3417 (14)	C22'—H22'	0.9500
C2'—C8'	1.4317 (15)	C23'—H23'	0.9500
N3'—C3A'	1.3830 (16)	C24'—H24'	0.9500
C3A'—C7A'	1.3898 (19)	C25'—H25'	0.9500
C3A'—C4'	1.3963 (17)	C26'—H26'	0.9500
C4'—C5'	1.382 (2)	N31—H031	0.86 (2)
C5'—C6'	1.388 (3)	N31—H032	0.87 (2)
C6'—C7'	1.388 (2)	C32—H32A	0.9900
C7'—C7A'	1.3967 (18)	C32—H32B	0.9900
C8'—C12'	1.4182 (14)	C33—H33A	0.9900
C8'—C9'	1.4536 (14)	C33—H33B	0.9900
C9'—O1'	1.2523 (13)	C34—H34A	0.9900
C9'—N1'	1.3739 (15)	C34—H34B	0.9900
C10'—O2'	1.2365 (13)	C35—H35A	0.9900
C10'—N1'	1.3784 (14)	C35—H35B	0.9900
C10'—C11'	1.4461 (13)	C36—H36A	0.9900
C11'—C12'	1.3821 (14)	C36—H36B	0.9900
C11'—C13'	1.4245 (14)		
			
C7A—S1—C2	89.05 (5)	C25'—C26'—C21'	119.68 (10)
N3—C2—C8	124.75 (9)	C32—N31—C36	113.41 (10)
N3—C2—S1	114.49 (8)	N31—C32—C33	110.43 (14)
C8—C2—S1	120.73 (7)	C32—C33—C34	112.44 (13)
C2—N3—C3A	111.53 (9)	C35—C34—C33	110.23 (12)
N3—C3A—C4	125.27 (10)	C34—C35—C36	111.26 (13)
N3—C3A—C7A	114.97 (10)	N31—C36—C35	110.53 (12)
C4—C3A—C7A	119.75 (10)	C5—C4—H4	120.7
C5—C4—C3A	118.64 (12)	C3A—C4—H4	120.7
C4—C5—C6	121.01 (13)	C4—C5—H5	119.5
C7—C6—C5	120.98 (11)	C6—C5—H5	119.5
C6—C7—C7A	118.48 (12)	C7—C6—H6	119.5
C7—C7A—C3A	121.13 (12)	C5—C6—H6	119.5
C7—C7A—S1	128.89 (10)	C6—C7—H7	120.8
C3A—C7A—S1	109.95 (8)	C7A—C7—H7	120.8
C12—C8—C9	118.71 (9)	C10—N1—H01	117.7 (11)
C12—C8—C2	124.59 (9)	C9—N1—H01	116.2 (11)
C9—C8—C2	116.60 (9)	C21—C22—H22	119.8
O1—C9—N1	118.08 (9)	C23—C22—H22	119.8
O1—C9—C8	124.20 (10)	C24—C23—H23	119.9
N1—C9—C8	117.71 (9)	C22—C23—H23	119.9
O2—C10—N1	119.22 (9)	C23—C24—H24	120.1
O2—C10—C11	124.96 (9)	C25—C24—H24	120.1
N1—C10—C11	115.79 (9)	C24—C25—H25	119.8
C12—C11—C13	123.00 (8)	C26—C25—H25	119.8
C12—C11—C10	121.26 (9)	C25—C26—H26	119.9
C13—C11—C10	115.73 (9)	C21—C26—H26	119.9
C8—C12—C11	120.09 (8)	C2'—N3'—H03'	117.0 (13)
C8—C12—C21	122.60 (8)	C3A'—N3'—H03'	127.0 (13)
C11—C12—C21	117.25 (8)	C5'—C4'—H4'	121.4
N2—C13—C11	176.22 (11)	C3A'—C4'—H4'	121.4
C10—N1—C9	126.18 (9)	C4'—C5'—H5'	119.3
C22—C21—C26	119.28 (9)	C6'—C5'—H5'	119.3
C22—C21—C12	120.73 (9)	C5'—C6'—H6'	119.1
C26—C21—C12	119.92 (9)	C7'—C6'—H6'	119.1
C21—C22—C23	120.32 (10)	C6'—C7'—H7'	121.4
C24—C23—C22	120.19 (11)	C7A'—C7'—H7'	121.4
C23—C24—C25	119.78 (10)	C9'—N1'—H01'	117.9 (11)
C24—C25—C26	120.31 (11)	C10'—N1'—H01'	115.6 (11)
C25—C26—C21	120.10 (10)	C23'—C22'—H22'	120.2
C2'—S1'—C7A'	91.12 (6)	C21'—C22'—H22'	120.2
N3'—C2'—C8'	121.35 (10)	C24'—C23'—H23'	119.8
N3'—C2'—S1'	110.55 (8)	C22'—C23'—H23'	119.8
C8'—C2'—S1'	128.07 (8)	C23'—C24'—H24'	120.1
C2'—N3'—C3A'	116.07 (11)	C25'—C24'—H24'	120.1
N3'—C3A'—C7A'	111.40 (10)	C26'—C25'—H25'	119.8
N3'—C3A'—C4'	126.91 (13)	C24'—C25'—H25'	119.8
C7A'—C3A'—C4'	121.68 (13)	C25'—C26'—H26'	120.2
C5'—C4'—C3A'	117.17 (15)	C21'—C26'—H26'	120.2
C4'—C5'—C6'	121.38 (13)	C32—N31—H031	106.4 (13)
C5'—C6'—C7'	121.77 (14)	C36—N31—H031	113.3 (13)
C6'—C7'—C7A'	117.18 (15)	C32—N31—H032	111.6 (13)
C3A'—C7A'—C7'	120.81 (12)	C36—N31—H032	109.8 (13)
C3A'—C7A'—S1'	110.84 (9)	H031—N31—H032	101.8 (17)
C7'—C7A'—S1'	128.34 (11)	N31—C32—H32A	109.6
C12'—C8'—C2'	123.78 (9)	C33—C32—H32A	109.6
C12'—C8'—C9'	118.97 (9)	N31—C32—H32B	109.6
C2'—C8'—C9'	117.24 (9)	C33—C32—H32B	109.6
O1'—C9'—N1'	117.97 (9)	H32A—C32—H32B	108.1
O1'—C9'—C8'	124.76 (10)	C32—C33—H33A	109.1
N1'—C9'—C8'	117.26 (9)	C34—C33—H33A	109.1
O2'—C10'—N1'	121.15 (9)	C32—C33—H33B	109.1
O2'—C10'—C11'	123.54 (10)	C34—C33—H33B	109.1
N1'—C10'—C11'	115.31 (9)	H33A—C33—H33B	107.8
C12'—C11'—C13'	123.09 (9)	C35—C34—H34A	109.6
C12'—C11'—C10'	122.12 (9)	C33—C34—H34A	109.6
C13'—C11'—C10'	114.74 (9)	C35—C34—H34B	109.6
C11'—C12'—C8'	120.01 (9)	C33—C34—H34B	109.6
C11'—C12'—C21'	119.01 (8)	H34A—C34—H34B	108.1
C8'—C12'—C21'	120.90 (9)	C34—C35—H35A	109.4
N2'—C13'—C11'	175.93 (11)	C36—C35—H35A	109.4
C9'—N1'—C10'	126.23 (9)	C34—C35—H35B	109.4
C22'—C21'—C26'	120.06 (9)	C36—C35—H35B	109.4
C22'—C21'—C12'	121.52 (8)	H35A—C35—H35B	108.0
C26'—C21'—C12'	118.42 (8)	N31—C36—H36A	109.5
C23'—C22'—C21'	119.51 (9)	C35—C36—H36A	109.5
C24'—C23'—C22'	120.42 (10)	N31—C36—H36B	109.5
C23'—C24'—C25'	119.86 (10)	C35—C36—H36B	109.5
C26'—C25'—C24'	120.45 (10)	H36A—C36—H36B	108.1
			
C7A—S1—C2—N3	−0.77 (8)	S1'—C2'—N3'—C3A'	1.61 (12)
C7A—S1—C2—C8	−178.73 (8)	C2'—N3'—C3A'—C7A'	−1.08 (14)
C8—C2—N3—C3A	178.49 (9)	C2'—N3'—C3A'—C4'	177.27 (11)
S1—C2—N3—C3A	0.62 (11)	N3'—C3A'—C4'—C5'	−178.06 (12)
C2—N3—C3A—C4	−178.93 (10)	C7A'—C3A'—C4'—C5'	0.13 (18)
C2—N3—C3A—C7A	−0.08 (13)	C3A'—C4'—C5'—C6'	−0.79 (19)
N3—C3A—C4—C5	179.44 (11)	C4'—C5'—C6'—C7'	1.2 (2)
C7A—C3A—C4—C5	0.64 (16)	C5'—C6'—C7'—C7A'	−0.95 (19)
C3A—C4—C5—C6	−0.72 (19)	N3'—C3A'—C7A'—C7'	178.56 (10)
C4—C5—C6—C7	0.0 (2)	C4'—C3A'—C7A'—C7'	0.11 (17)
C5—C6—C7—C7A	0.8 (2)	N3'—C3A'—C7A'—S1'	0.03 (12)
C6—C7—C7A—C3A	−0.85 (18)	C4'—C3A'—C7A'—S1'	−178.42 (9)
C6—C7—C7A—S1	−178.78 (10)	C6'—C7'—C7A'—C3A'	0.29 (17)
N3—C3A—C7A—C7	−178.78 (10)	C6'—C7'—C7A'—S1'	178.54 (9)
C4—C3A—C7A—C7	0.14 (16)	C2'—S1'—C7A'—C3A'	0.71 (8)
N3—C3A—C7A—S1	−0.49 (12)	C2'—S1'—C7A'—C7'	−177.68 (11)
C4—C3A—C7A—S1	178.43 (8)	N3'—C2'—C8'—C12'	175.67 (9)
C2—S1—C7A—C7	178.78 (11)	S1'—C2'—C8'—C12'	−6.52 (15)
C2—S1—C7A—C3A	0.67 (8)	N3'—C2'—C8'—C9'	−4.04 (14)
N3—C2—C8—C12	11.74 (15)	S1'—C2'—C8'—C9'	173.77 (7)
S1—C2—C8—C12	−170.52 (7)	C12'—C8'—C9'—O1'	−177.30 (9)
N3—C2—C8—C9	−164.55 (9)	C2'—C8'—C9'—O1'	2.43 (15)
S1—C2—C8—C9	13.19 (12)	C12'—C8'—C9'—N1'	3.46 (13)
C12—C8—C9—O1	175.35 (10)	C2'—C8'—C9'—N1'	−176.80 (9)
C2—C8—C9—O1	−8.13 (15)	O2'—C10'—C11'—C12'	178.63 (10)
C12—C8—C9—N1	−3.82 (13)	N1'—C10'—C11'—C12'	−0.63 (14)
C2—C8—C9—N1	172.70 (9)	O2'—C10'—C11'—C13'	1.01 (15)
O2—C10—C11—C12	179.76 (10)	N1'—C10'—C11'—C13'	−178.25 (9)
N1—C10—C11—C12	−2.32 (14)	C13'—C11'—C12'—C8'	179.62 (9)
O2—C10—C11—C13	−1.57 (15)	C10'—C11'—C12'—C8'	2.20 (14)
N1—C10—C11—C13	176.35 (9)	C13'—C11'—C12'—C21'	2.99 (14)
C9—C8—C12—C11	5.22 (13)	C10'—C11'—C12'—C21'	−174.43 (9)
C2—C8—C12—C11	−170.99 (9)	C2'—C8'—C12'—C11'	176.69 (9)
C9—C8—C12—C21	−172.04 (9)	C9'—C8'—C12'—C11'	−3.60 (14)
C2—C8—C12—C21	11.75 (14)	C2'—C8'—C12'—C21'	−6.75 (14)
C13—C11—C12—C8	179.29 (9)	C9'—C8'—C12'—C21'	172.96 (8)
C10—C11—C12—C8	−2.15 (14)	O1'—C9'—N1'—C10'	178.65 (10)
C13—C11—C12—C21	−3.31 (14)	C8'—C9'—N1'—C10'	−2.06 (15)
C10—C11—C12—C21	175.25 (9)	O2'—C10'—N1'—C9'	−178.66 (10)
O2—C10—N1—C9	−178.05 (10)	C11'—C10'—N1'—C9'	0.62 (14)
C11—C10—N1—C9	3.90 (15)	C11'—C12'—C21'—C22'	−81.65 (12)
O1—C9—N1—C10	179.90 (10)	C8'—C12'—C21'—C22'	101.76 (11)
C8—C9—N1—C10	−0.87 (15)	C11'—C12'—C21'—C26'	99.31 (11)
C8—C12—C21—C22	−125.55 (10)	C8'—C12'—C21'—C26'	−77.29 (12)
C11—C12—C21—C22	57.12 (12)	C26'—C21'—C22'—C23'	1.92 (15)
C8—C12—C21—C26	57.61 (13)	C12'—C21'—C22'—C23'	−177.11 (9)
C11—C12—C21—C26	−119.72 (10)	C21'—C22'—C23'—C24'	−1.15 (16)
C26—C21—C22—C23	−1.44 (15)	C22'—C23'—C24'—C25'	−0.25 (17)
C12—C21—C22—C23	−178.30 (9)	C23'—C24'—C25'—C26'	0.91 (18)
C21—C22—C23—C24	1.10 (17)	C24'—C25'—C26'—C21'	−0.13 (17)
C22—C23—C24—C25	0.06 (17)	C22'—C21'—C26'—C25'	−1.28 (15)
C23—C24—C25—C26	−0.87 (17)	C12'—C21'—C26'—C25'	177.78 (10)
C24—C25—C26—C21	0.52 (16)	C36—N31—C32—C33	−54.46 (16)
C22—C21—C26—C25	0.63 (15)	N31—C32—C33—C34	53.39 (19)
C12—C21—C26—C25	177.52 (9)	C32—C33—C34—C35	−54.42 (19)
C7A'—S1'—C2'—N3'	−1.29 (8)	C33—C34—C35—C36	55.39 (17)
C7A'—S1'—C2'—C8'	−179.30 (9)	C32—N31—C36—C35	56.13 (16)
C8'—C2'—N3'—C3A'	179.77 (9)	C34—C35—C36—N31	−56.07 (16)

### Piperidinium (Z)-5-(benzo[d]thiazol-2-yl)-3-cyano-6-oxo-4-phenyl-1,6-dihydropyridin-2-olate–(E)-5-(2,3-dihydrobenzo[d]thiazol-2-ylidene)-2,6-dioxo-4-phenyl-1,2,5,6-tetrahydropyridine-3-carbonitrile (1/1) Hydrogen-bond geometry (Å, º)

**Table:**

*D*—H···*A*	*D*—H	H···*A*	*D*···*A*	*D*—H···*A*
N1—H01···O2′	0.888 (18)	1.887 (18)	2.7703 (11)	172.9 (16)
N3′—H03′···O1′	0.804 (19)	1.878 (19)	2.5399 (15)	139.0 (18)
N1′—H01′···O2	0.883 (17)	2.016 (18)	2.8903 (11)	169.8 (16)
N31—H031···O1	0.86 (2)	1.91 (2)	2.7327 (12)	158.7 (18)
N31—H032···O2^i^	0.87 (2)	1.88 (2)	2.7322 (13)	169.3 (19)
C36—H36*B*···O1′^i^	0.99	2.52	3.4487 (16)	157
C22′—H22′···O1′^ii^	0.95	2.34	3.2691 (13)	165
C26′—H26′···N3^i^	0.95	2.44	3.3437 (13)	159

Symmetry codes: (i) −*x*+1, −*y*+1, −*z*+1; (ii) −*x*, −*y*+1, −*z*+1.
